# Supplementary material for: Quality Attributes and Metabolic Profiles of Uvaia (Eugenia pyriformis), a Native Brazilian Atlantic Forest Fruit
Source: Foods. 2023 May 3;12(9):1881. doi: 10.3390/foods12091881 (PMC10177832; doi:10.3390/foods12091881)
Supplement: Supplementary file 1 [file foods-12-01881-s001.zip › foods-2242362-supplementary.pdf]

foods

Article

Quality Attributes and Metabolic Profiles of Uvaia (*Eugenia pyriformis*), a Native Brazilian Atlantic Forest Fruit

Poliana Cristina Spricigo [1,2], Luísa Souza Almeida [3], Gabriel Henrique Ribeiro [4], Banny Silva Barbosa Correia [3,5], Isabela Barroso Taver [1], Angelo Pedro Jacomino [1,*] and Luiz Alberto Colnago [4]

[1] Luiz de Queiroz College of Agriculture, University of São Paulo, 11 Pádua Dias Ave., Piracicaba 13418-900, São Paulo, Brazil; polianaspricigo@usp.br (P.C.S.)
[2] School of Agricultural and Veterinarian Sciences, São Paulo State University, Jaboticabal 14884-900, São Paulo, Brazil
[3] Institute of Chemistry of São Carlos, University of São Paulo, 400 Trabalhador São Carlense Ave., São Carlos 13566-590, São Paulo, Brazil
[4] Embrapa Instrumentation, 1452 XV de Novembro Street, São Carlos 13560-970, São Paulo, Brazil; luiz.colnago@embrapa.br (L.A.C.)
[5] Department of Food Science, Aarhus University, 48 Agro Food Park, Aarhus 8200, Jutland, Denmark
* Correspondence: jacomino@usp.br

Supporting Information

Table S1. Physicochemical uvaia characteristics– 2020 harvest.

	Accession	*Height (mm)*	*Diameter. (mm)*	*Fresh mass (g)*	*Seed mass (g)*	*Yield.*	*TSS*	*TTA*
1	UV016	16.21 ± 0.16	20.83 ± 0.77	4.80 ± 0.23	1.26 ± 0.20	74.13 ± 2.62	10.08 ± 0.60	1.69 ± 0.12
2	UV022	24.83 ± 0.89	30.22 ± 2.16	13.85 ± 0.56	4.26 ± 0.49	70.91 ± 2.42	8.12 ± 0.35	1.48 ± 0.03
3	UV027	17.96 ± 0.62	21.11 ± 0.34	5.41 ± 0.15	1.19 ± 0.13	78.30 ± 2.00	12.73 ± 0.33	2.08 ± 0.09
4	UV028	22.08 ± 0.07	26.18 ± 0.40	8.91 ± 0.13	1.34 ± 0.13	85.07 ± 1.24	15.30 ± 0.46	1.39 ± 0.22
5	UV029	26.38 ± 0.62	33.82 ± 0.70	14.18 ± 0.71	3.46 ± 0.86	75.94 ± 6.11	7.52 ± 0.21	1.25 ± 0.30
6	UV030	20.67 ± 0.59	25.09 ± 0.07	12.88 ± 2.17	1.24 ± 0.06	89.95 ± 1.19	13.92 ± 0.56	1.00 ± 0.14
7	UV036	19.20 ± 0.58	19.51 ± 0.19	4.29 ± 0.27	1.26 ± 0.12	70.79 ± 1.62	8.73 ± 0.34	1.10 ± 0.11
8	UV037	24.11 ± 0.34	28.03 ± 0.18	10.28 ± 0.54	2.48 ± 0.12	75.92 ± 1.80	8.40 ± 0.13	1.06 ± 0.11
9	UV039	20.30 ± 0.60	25.51 ± 1.03	6.83 ± 0.26	1.10 ± 0.05	84.04 ± 1.16	6.57 ± 0.13	0.95 ± 0.09
10	UV040	24.99 ± 0.98	30.36 ± 0.63	11.64 ± 0.48	1.73 ± 0.16	85.58 ± 1.35	12.07 ± 0.40	1.29 ± 0.08
11	UV041	23.12 ± 0.68	25.80 ± 0.35	9.30 ± 0.30	2.89 ± 0.20	69.24 ± 2.54	8.42 ± 0.30	1.36 ± 0.22
12	UV042	19.92 ± 1.02	24.05 ± 0.25	6.51 ± 0.27	1.15 ± 0.08	82.53 ± 0.79	9.10 ± 0.26	1.22 ± 0.02
13	UV043	20.92 ± 0.70	25.50 ± 0.30	6.65 ± 0.77	1.48 ± 0.14	64.87 ± 25.7	8.72 ± 0.48	1.10 ± 0.36
14	UV046	21.94 ± 1.49	23.69 ± 1.66	7.44 ± 0.95	2.26 ± 0.62	70.72 ± 4.23	10.02 ± 0.21	2.02 ± 0.13
15	UV047	23.82 ± 0.78	27.04 ± 0.59	9.92 ± 0.70	1.56 ± 0.32	84.06 ± 3.62	12.30 ± 0.83	1.11 ± 0.11
16	UV048	18.59 ± 0.49	21.65 ± 0.52	5.55 ± 0.82	1.40 ± 0.21	74.60 ± 2.32	10.27 ± 0.70	2.31 ± 0.19
17	UV049	26.16 ± 0.65	30.21 ± 0.25	13.14 ± 0.27	3.18 ± 0.03	76.35 ± 0.21	8.95 ± 0.35	2.26 ± 0.13
18	UV050	20.49 ± 0.46	23.67 ± 0.98	6.54 ± 0.54	2.15 ± 0.93	65.95 ± 1.05	8.62 ± 0.36	1.47 ± 0.09
19	UV052	25.56 ± 1.33	31.99 ± 0.32	12.92 ± 0.28	2.42 ± 0.13	81.39 ± 0.76	7.28 ± 0.24	0.83 ± 0.39
20	UV056	21.50 ± 1.58	24.65 ± 0.86	7.75 ± 0.85	1.50 ± 0.10	80.76 ± 1.10	9.12 ± 0.43	1.47 ± 0.07
21	UV057	20.19 ± 0.63	26.24 ± 0.04	7.36 ± 0.36	1.14 ± 0.14	84.70 ± 1.26	8.13 ± 0.15	1.18 ± 0.03
22	UV058	24.97 ± 1.58	28.03 ± 1.70	12.46 ± 1.41	3.45 ± 0.72	73.51 ± 2.25	7.58 ± 0.46	1.27 ± 0.29
23	UV059	20.09 ± 0.54	24.90 ± 0.57	7.46 ± 0.19	1.53 ± 0.25	79.65 ± 3.43	8.98 ± 0.23	1.38 ± 0.27
24	UV060	20.00 ± 0.44	23.19 ± 1.31	6.81 ± 0.57	1.15 ± 0.11	83.10 ± 0.22	11.38 ± 0.25	2.07 ± 0.07

25	UV062	28.17 ± 0.66	32.03 ± 6.42	26.23 ± 7.27	3.96 ± 1.27	85.60 ± 1.05	10.43 ± 0.32	1.69 ± 0.03
26	UV067	19.81 ± 0.72	23.88 ± 0.79	7.61 ± 0.22	1.06 ± 0.14	86.20 ± 1.72	10.20 ± 0.23	1.49 ± 0.08
27	UV068	32.62 ± 0.80	41.17 ± 0.74	27.90 ± 1.61	5.47 ± 1.45	80.47 ± 6.67	10.20 ± 0.17	1.03 ± 0.11
28	UV069	21.39 ± 1.12	24.00 ± 0.33	8.08 ± 0.65	2.58 ± 0.27	69.27 ± 2.81	10.45 ± 0.13	3.48 ± 0.04
29	UV072	19.10 ± 0.39	24.97 ± 0.37	6.59 ± 0.29	1.82 ± 0.21	72.99 ± 1.16	5.98 ± 0.25	1.77 ± 0.04
30	UV073	30.09 ± 0.98	34.94 ± 0.71	18.19 ± 1.62	3.02 ± 0.19	83.45 ± 0.71	10.53 ± 0.38	3.46 ± 0.04
31	UV075	26.74 ± 0.60	31.25 ± 0.46	13.63 ± 0.69	4.05 ± 0.10	70.33 ± 0.76	10.38 ± 0.57	3.13 ± 0.02
32	UV076	20.06 ± 0.45	24.59 ± 1.01	6.79 ± 0.48	1.91 ± 0.38	72.49 ± 3.85	8.77 ± 0.58	3.58 ± 0.04
33	UV088	24.05 ± 0.50	28.82 ± 0.26	11.12 ± 0.09	4.24 ± 0.57	61.95 ± 4.94	5.87 ± 0.21	3.17 ± 0.02
34	UV089	21.40 ± 0.31	26.93 ± 0.48	7.40 ± 0.40	1.54 ± 0.10	79.37 ± 1.66	9.50 ± 0.71	3.08 ± 0.08
35	UV112	22.11 ± 0.35	26.91 ± 0.61	9.22 ± 0.22	1.60 ± 0.16	82.65 ± 2.13	8.92 ± 0.32	2.85 ± 0.03
36	UV114	20.03 ± 0.66	24.39 ± 1.01	6.64 ± 0.18	0.84 ± 0.16	87.59 ± 1.49	10.17 ± 0.42	3.16 ± 0.07
37	UV116	22.79 ± 1.06	29.55 ± 1.05	13.81 ± 0.36	2.72 ± 0.55	80.37 ± 4.55	9.55 ± 0.28	3.01 ± 0.19
38	UV120	20.50 ± 0.50	24.19 ± 0.72	7.45 ± 0.24	1.47 ± 0.08	80.79 ± 0.64	9.05 ± 0.22	2.87 ± 0.05
39	UV122	22.42 ± 0.61	24.91 ± 0.50	9.37 ± 0.75	2.08 ± 0.07	77.09 ± 3.02	9.78 ± 0.32	1.65 ± 0.02
40	UV125	21.40 ± 0.56	24.24 ± 0.45	6.85 ± 0.25	1.26 ± 0.12	81.58 ± 1.39	10.28 ± 0.20	3.70 ± 0.06
41	UV146	25.62 ± 0.90	28.14 ± 1.40	10.49 ± 0.62	1.81 ± 0.08	82.59 ± 1.64	11.17 ± 0.23	3.13 ± 0.05

Accession identification codes: UV = uvaia, three-digit number = accession identification. Results expressed as means + standard deviations. TSS: total soluble solids (°Brix); TTA: total titratable acidity (% citric acid eq).

Table S2. 2D structure of identified uvaia metabolites.

Sugar and related compounds	***Acids and related compounds***	***Aminoacids and related compounds***
Sucrose 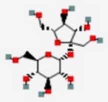	*Citrate* 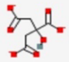	*Alanine* 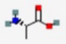
Glucose 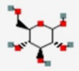	*Malate* 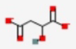	*Arginine* 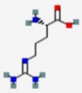
Fructose 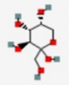	*Gallate* 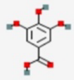	*Asparagine* 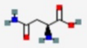
Ethanol	*Succinate* 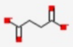	*Choline* 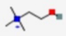
Methanol	*4-Aminobutyrate* 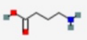	*Glutamine* 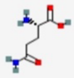
Uridine 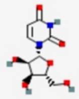	*Cinnamate* 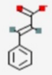	*Isoleucine* 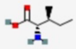
	Formate 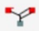	*Leucine* 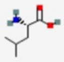
	Acetamide 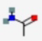	*Phenylalanine* 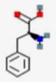
		Proline 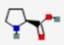
		Serine 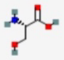
		Threonine 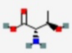
		Tyrosine 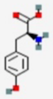
		Valine 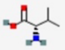

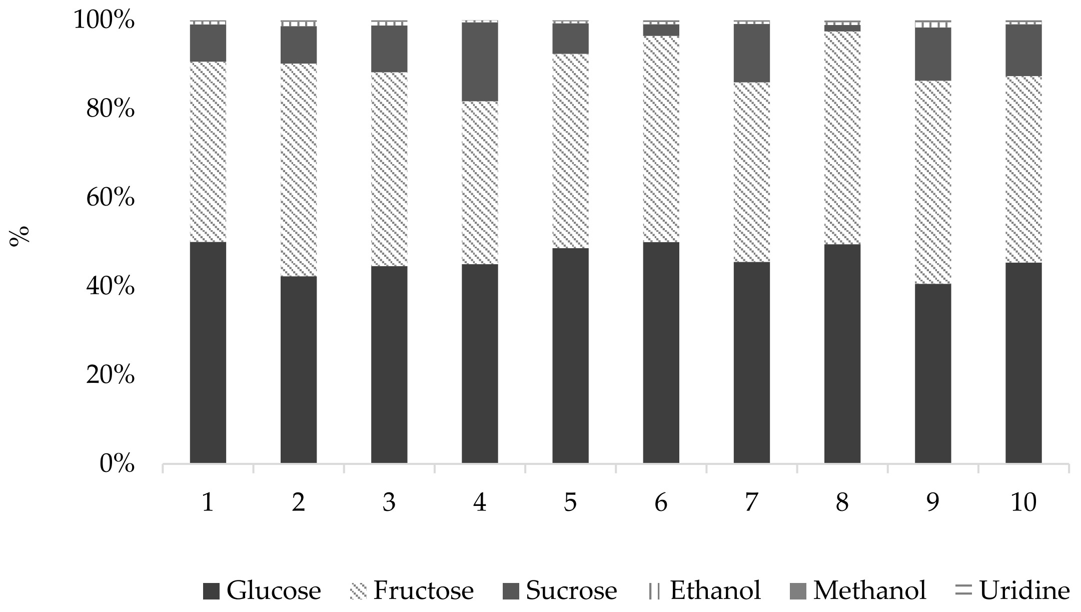

Figure S1. Sugar and related compounds content found in 10 selected uvaia accessions.

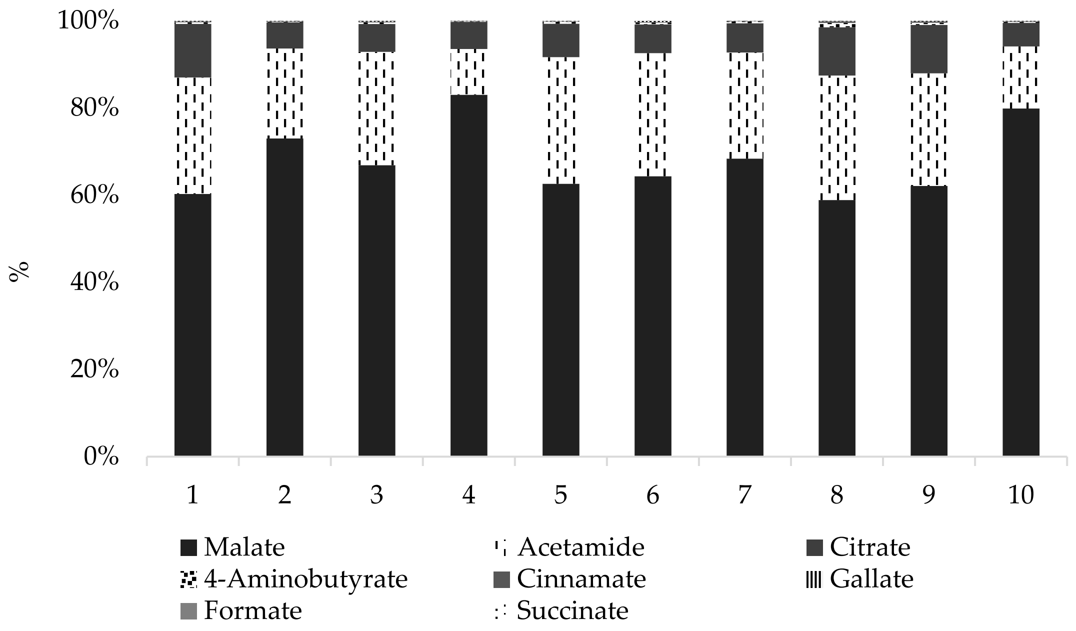

Figure S2. Acids and related compounds content found in 10 selected uvaia accessions.

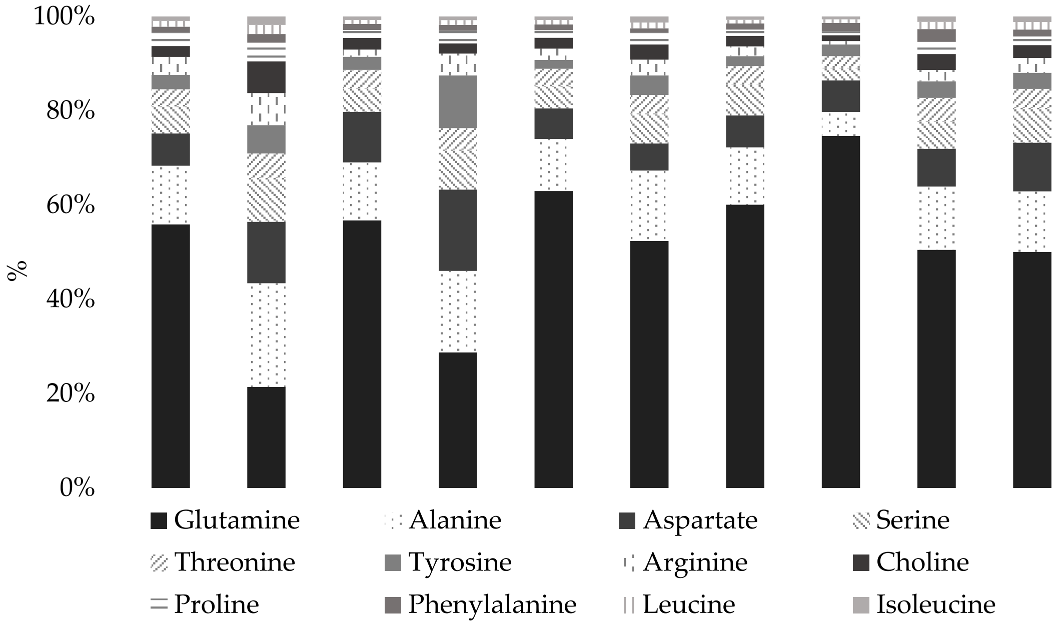

Figure S3. Amino acids and related compounds content found in 10 selected uvaia accessions.

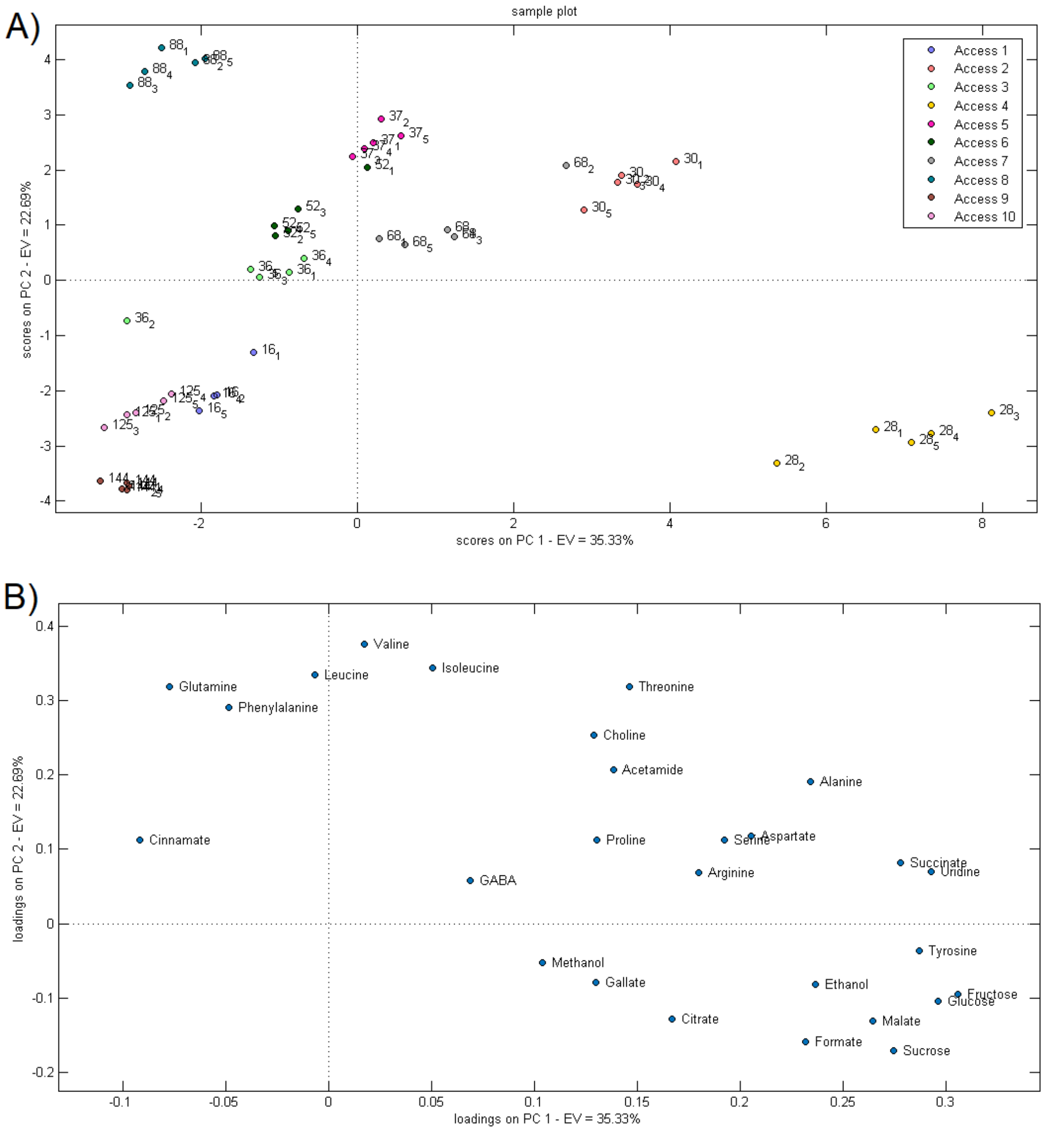

Figure S4. Principal component analysis (A) scores and (B) loadings concerning uvaia quality attributes according to metabolites identified by NMR.

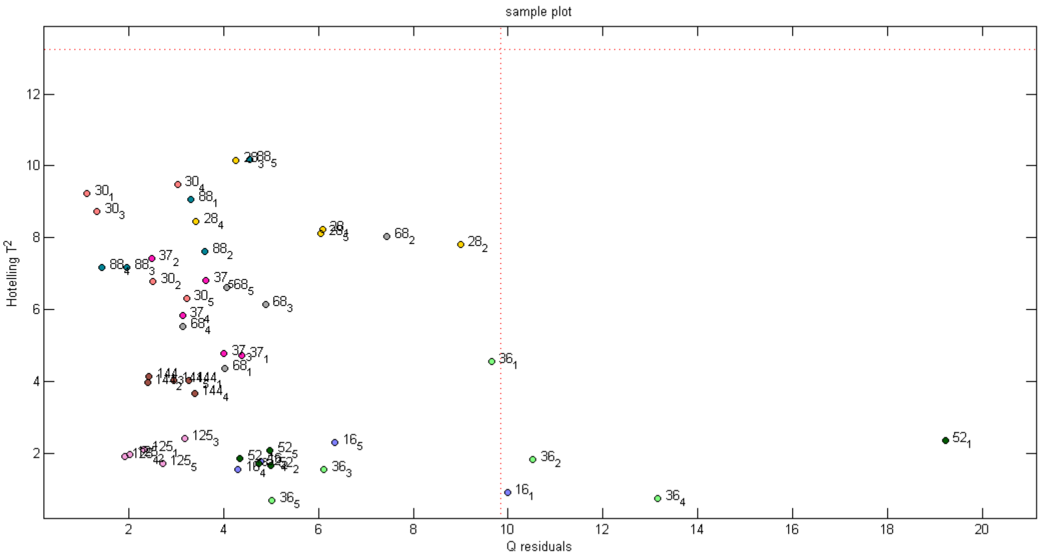

Figure S5. Plot of Q residuals x Hotelling of T^2. Indicates whether the samples grouped in the PCA have residuals (high values on the x axis) which would indicate that they were not well explained by the PCs and also specify whether they are samples with more than 2 standard deviations (y axis) deviating from the characteristics of the larger group. Samples presenting high residue and T^2 values are considered outliers.

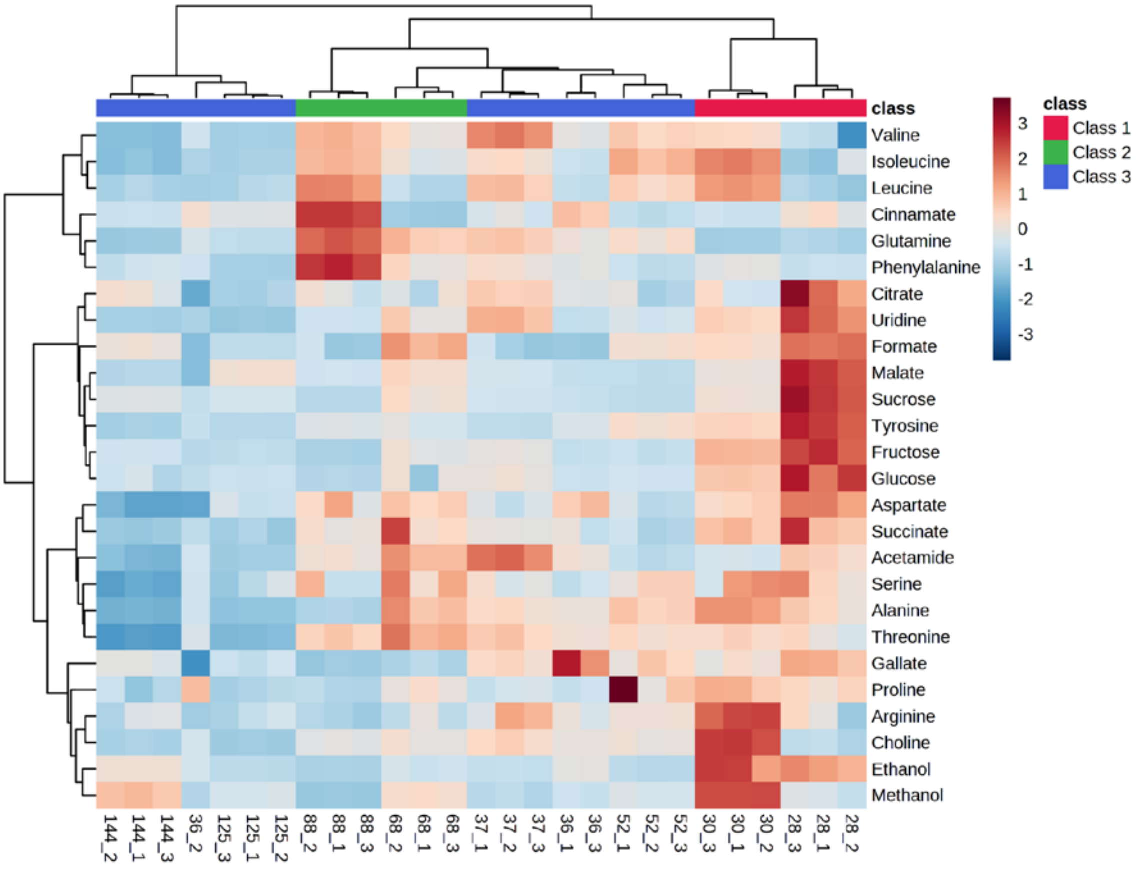

Figure S6. Class heatmaps. Class 1: SS, Y Accessions 2, and 4; Class 2: S, FW, D and H Accessions 7 and 8; Class 3: TA Accessions 3, 5, 6, 9, 10.

Table S3. Uvaia classifications according to their physicochemical characteristics - Class 1: SS, Y. Accession 2, and 4.

		Total Cmpd	Hits	Raw p	_-LOG10(p)	Holm adjust	FDR	Impact
1	Isoquinoline alkaloid biosynthesis	6	1	4.10E-07	6.3869	1.48E-05	2.61E-06	0.5
2	Phenylalanine metabolism	11	1	0.3835	0.41624	1	0.41637	0.47059
3	Alanine, aspartate and glutamate metabolism	22	4	6.67E-05	4.1761	0.0017335	0.00017179	0.32014
4	Citrate cycle (TCA cycle)	20	3	6.78E-05	4.1687	0.0017335	0.00017179	0.18889
5	Arginine and proline metabolism	34	2	0.003544	2.4505	0.048245	0.0051797	0.17461
6	Pyruvate metabolism	22	1	6.75E-05	4.1706	0.0017335	0.00017179	0.15462
7	Glycine, serine and threonine metabolism	33	3	0.0061796	2.209	0.074155	0.0086972	0.1204
8	Tyrosine metabolism	16	1	4.10E-07	6.3869	1.48E-05	2.61E-06	0.10811
9	Starch and sucrose metabolism	22	1	1.14E-05	4.9427	0.00033088	3.94E-05	0.0889
10	Arginine biosynthesis	18	3	1.37E-06	5.863	4.39E-05	7.44E-06	0.08447
11	Glyoxylate and dicarboxylate metabolism	29	5	6.46E-06	5.1898	0.00019381	2.73E-05	0.06741
12	Carbon fixation in photosynthetic organisms	21	3	5.18E-05	4.2856	0.0013988	0.00016406	0.05846
13	Galactose metabolism	27	1	1.14E-05	4.9427	0.00033088	3.94E-05	0.04252
14	Sulfur metabolism	15	1	0.00098609	3.0061	0.020708	0.0018736	0.03315
15	Pyrimidine metabolism	38	2	4.12E-07	6.3849	1.48E-05	2.61E-06	0.03089
16	Glycerophospholipid metabolism	37	1	0.012865	1.8906	0.10292	0.01577	0.03075
17	Phenylalanine, tyrosine and tryptophan biosynthesis	22	2	3.08E-06	5.5118	9.54E-05	1.46E-05	0.02152
18	Glycolysis / Gluconeogenesis	26	2	4.07E-14	13.39	1.55E-12	1.55E-12	0.00038
19	Amino sugar and nucleotide sugar metabolism	50	1	1.97E-10	9.7049	7.30E-09	3.75E-09	0
20	Ubiquinone and other terpenoid-quinone biosynthesis	38	1	4.10E-07	6.3869	1.48E-05	2.61E-06	0
21	Nitrogen metabolism	12	2	0.00016489	3.7828	0.0037926	0.00039162	0
22	Aminoacyl-tRNA biosynthesis	46	11	0.00064081	3.1933	0.014098	0.0014324	0
23	Propanoate metabolism	20	1	0.00098609	3.0061	0.020708	0.0018736	0
24	Butanoate metabolism	17	1	0.00098609	3.0061	0.020708	0.0018736	0
25	Monobactam biosynthesis	8	1	0.0026803	2.5718	0.048245	0.0040741	0
26	Cysteine and methionine metabolism	46	1	0.0026803	2.5718	0.048245	0.0040741	0
27	Lysine biosynthesis	9	1	0.0026803	2.5718	0.048245	0.0040741	0

28	beta-Alanine metabolism	18	1	0.0026803	2.5718	0.048245	0.0040741	0
29	Nicotinate and nicotinamide metabolism	13	1	0.0026803	2.5718	0.048245	0.0040741	0
30	Purine metabolism	63	1	0.0070533	2.1516	0.077586	0.0095723	0
31	Cyanoamino acid metabolism	29	2	0.0095249	2.0211	0.095249	0.012481	0
32	Selenocompound metabolism	13	1	0.01013	1.9944	0.095249	0.012832	0
33	Pantothenate and CoA biosynthesis	23	1	0.32916	0.4826	1	0.39087	0
34	Phenylpropanoid biosynthesis	46	1	0.3835	0.41624	1	0.41637	0
35	Tropane, piperidine and pyridine alkaloid biosynthesis	8	1	0.3835	0.41624	1	0.41637	0
36	Valine, leucine and isoleucine degradation	37	3	0.40154	0.39627	1	0.42385	0
37	Glucosinolate biosynthesis	65	4	0.42835	0.3682	1	0.43993	0
38	Valine, leucine and isoleucine biosynthesis	22	4	0.46672	0.33094	1	0.46672	0

Table S4. Table S3. Uvaia classifications according to their physicochemical characteristics - Class 2: S, FW, D and H. Accession 7 and 8.

		Total Cmpd	Hits	Raw p	_-LOG10(p)	Holm adjust	FDR	Impact
1	Isoquinoline alkaloid biosynthesis	6	1	0.52673	0.27841	1	0.57188	0.5
2	Phenylalanine metabolism	11	1	9.20E-06	5.0364	0.00034026	7.18E-05	0.47059
3	Alanine, aspartate and glutamate metabolism	22	4	0.0034762	2.4589	0.097334	0.012009	0.32014
4	Citrate cycle (TCA cycle)	20	3	0.36108	0.4424	1	0.47314	0.18889
5	Arginine and proline metabolism	34	2	0.10968	0.95987	1	0.20661	0.17461
6	Pyruvate metabolism	22	1	0.88676	0.052191	1	0.90561	0.15462
7	Glycine, serine and threonine metabolism	33	3	0.040386	1.3938	1	0.10962	0.1204
8	Tyrosine metabolism	16	1	0.52673	0.27841	1	0.57188	0.10811
9	Starch and sucrose metabolism	22	1	0.46412	0.33337	1	0.55739	0.0889
10	Arginine biosynthesis	18	3	9.45E-06	5.0247	0.00034026	7.18E-05	0.08447
11	Glyoxylate and dicarboxylate metabolism	29	5	0.018843	1.7249	0.48991	0.055079	0.06741
12	Carbon fixation in photosynthetic organisms	21	3	0.46938	0.32847	1	0.55739	0.05846
13	Galactose metabolism	27	1	0.46412	0.33337	1	0.55739	0.04252
14	Sulfur metabolism	15	1	0.12224	0.9128	1	0.20661	0.03315
15	Pyrimidine metabolism	38	2	4.35E-05	4.3612	0.0014367	0.00027427	0.03089
16	Glycerophospholipid metabolism	37	1	0.90561	0.043061	1	0.90561	0.03075
17	Phenylalanine, tyrosine and tryptophan biosynthesis	22	2	6.45E-05	4.1906	0.0019986	0.00030623	0.02152
18	Glycolysis / Gluconeogenesis	26	2	0.068613	1.1636	1	0.16296	0.00038
19	Purine metabolism	63	1	6.17E-06	5.21	0.00023431	7.18E-05	0
20	Phenylpropanoid biosynthesis	46	1	9.20E-06	5.0364	0.00034026	7.18E-05	0
21	Tropane, piperidine and pyridine alkaloid biosynthesis	8	1	9.20E-06	5.0364	0.00034026	7.18E-05	0
22	Cyanoamino acid metabolism	29	2	5.05E-05	4.2965	0.0016168	0.00027427	0
23	Nitrogen metabolism	12	2	0.00030299	3.5186	0.0090897	0.0012793	0
24	Aminoacyl-tRNA biosynthesis	46	11	0.0014055	2.8522	0.040759	0.0053408	0
25	Glucosinolate biosynthesis	65	4	0.010784	1.9672	0.29117	0.034149	0

26	Valine, leucine and isoleucine biosynthesis	22	4	0.060033	1.2216	1	0.15208	0
27	Propanoate metabolism	20	1	0.12224	0.9128	1	0.20661	0
28	Butanoate metabolism	17	1	0.12224	0.9128	1	0.20661	0
29	Pantothenate and CoA biosynthesis	23	1	0.13602	0.8664	1	0.20661	0
30	Monobactam biosynthesis	8	1	0.14136	0.84966	1	0.20661	0
31	Cysteine and methionine metabolism	46	1	0.14136	0.84966	1	0.20661	0
32	Lysine biosynthesis	9	1	0.14136	0.84966	1	0.20661	0
33	beta-Alanine metabolism	18	1	0.14136	0.84966	1	0.20661	0
34	Nicotinate and nicotinamide metabolism	13	1	0.14136	0.84966	1	0.20661	0
35	Amino sugar and nucleotide sugar metabolism	50	1	0.16641	0.77882	1	0.23421	0
36	Valine, leucine and isoleucine degradation	37	3	0.19649	0.70666	1	0.26667	0
37	Ubiquinone and other terpenoid-quinone biosynthesis	38	1	0.52673	0.27841	1	0.57188	0
38	Selenocompound metabolism	13	1	0.7951	0.099577	1	0.83928	0

Table S5. Table S3. Uvaia classifications according to their physicochemical characteristics - Class 3: TTA. Accessions 3,5,6,9,10.

		Total Cmpd	Hits	Raw p	_-LOG10(p)	Holm adjust	FDR	Impact
1	Isoquinoline alkaloid biosynthesis	6	1	0.0020404	2.6903	0.042848	0.0038767	0.5
2	Phenylalanine metabolism	11	1	0.012262	1.9114	0.14714	0.016067	0.47059
3	Alanine, aspartate and glutamate metabolism	22	4	1.04E-05	4.9817	0.00035467	7.93E-05	0.32014
4	Citrate cycle (TCA cycle)	20	3	0.00025308	3.5967	0.0060739	0.00064114	0.18889
5	Arginine and proline metabolism	34	2	0.57324	0.24166	1	0.58873	0.17461
6	Pyruvate metabolism	22	1	0.0027451	2.5614	0.049413	0.0049674	0.15462
7	Glycine, serine and threonine metabolism	33	3	8.29E-05	4.0814	0.0022385	0.00026254	0.1204
8	Tyrosine metabolism	16	1	0.0020404	2.6903	0.042848	0.0038767	0.10811
9	Starch and sucrose metabolism	22	1	0.0088299	2.054	0.13667	0.013421	0.0889
10	Arginine biosynthesis	18	3	0.00090591	3.0429	0.020836	0.0021515	0.08447
11	Glyoxylate and dicarboxylate metabolism	29	5	0.00018738	3.7273	0.0046844	0.0005086	0.06741
12	Carbon fixation in photosynthetic organisms	21	3	5.95E-05	4.2258	0.0016648	0.00020539	0.05846
13	Galactose metabolism	27	1	0.0088299	2.054	0.13667	0.013421	0.04252
14	Sulfur metabolism	15	1	5.27E-06	5.2782	0.00020026	6.31E-05	0.03315
15	Pyrimidine metabolism	38	2	0.014924	1.8261	0.14714	0.018904	0.03089
16	Glycerophospholipid metabolism	37	1	0.05386	1.2687	0.32316	0.06202	0.03075
17	Phenylalanine, tyrosine and tryptophan biosynthesis	22	2	0.00010548	3.9768	0.0027425	0.00030833	0.02152
18	Glycolysis / Gluconeogenesis	26	2	0.010472	1.98	0.13667	0.015305	0.00038
19	Propanoate metabolism	20	1	5.27E-06	5.2782	0.00020026	6.31E-05	0
20	Butanoate metabolism	17	1	5.27E-06	5.2782	0.00020026	6.31E-05	0
21	Cyanoamino acid metabolism	29	2	6.65E-06	5.1774	0.00023262	6.31E-05	0
22	Monobactam biosynthesis	8	1	3.72E-05	4.43	0.0012262	0.0001412	0
23	Cysteine and methionine metabolism	46	1	3.72E-05	4.43	0.0012262	0.0001412	0
24	Lysine biosynthesis	9	1	3.72E-05	4.43	0.0012262	0.0001412	0
25	beta-Alanine metabolism	18	1	3.72E-05	4.43	0.0012262	0.0001412	0
26	Nicotinate and nicotinamide metabolism	13	1	3.72E-05	4.43	0.0012262	0.0001412	0
27	Aminoacyl-tRNA biosynthesis	46	11	0.0014299	2.8447	0.031457	0.0031962	0

28	Ubiquinone and other terpenoid-quinone biosynthesis	38	1	0.0020404	2.6903	0.042848	0.0038767	0
29	Amino sugar and nucleotide sugar metabolism	50	1	0.0051765	2.286	0.088	0.0089411	0
30	Nitrogen metabolism	12	2	0.0085419	2.0684	0.13667	0.013421	0
31	Phenylpropanoid biosynthesis	46	1	0.012262	1.9114	0.14714	0.016067	0
32	Tropane, piperidine and pyridine alkaloid biosynthesis	8	1	0.012262	1.9114	0.14714	0.016067	0
33	Selenocompound metabolism	13	1	0.018326	1.7369	0.14714	0.022464	0
34	Valine, leucine and isoleucine biosynthesis	22	4	0.045305	1.3439	0.31713	0.053799	0
35	Glucosinolate biosynthesis	65	4	0.062311	1.2054	0.32316	0.069641	0
36	Valine, leucine and isoleucine degradation	37	3	0.16542	0.7814	0.66169	0.1796	0
37	Purine metabolism	63	1	0.30398	0.51715	0.91195	0.32087	0
38	Pantothenate and CoA biosynthesis	23	1	0.68062	0.1671	1	0.68062	0
